# A polymorphism rs3746444 within the pre‐miR‐499 alters the maturation of miR‐499‐5p and its antiapoptotic function

**DOI:** 10.1111/jcmm.13813

**Published:** 2018-08-13

**Authors:** Wei Ding, Mengyang Li, Teng Sun, Di Han, Xiaoci Guo, Xiao Chen, Qinggong Wan, Xuejuan Zhang, Jianxun Wang

**Affiliations:** ^1^ Department of General Medicine The Affiliated Hospital Qingdao University Qingdao China; ^2^ Institute for Translational Medicine Qingdao University Qingdao China

**Keywords:** apoptosis, heart failure, miR‐499‐5p, myocardial infarction, SNPs

## Abstract

microRNAs (miRNAs) are non‐coding RNAs that function as post‐transcriptional regulators of cardiac development and cardiovascular diseases. Single nucleotide polymorphisms (SNPs) in miRNA genes are a novel class of genetic variations in the human genome that confer the risk of cardiovascular diseases. Here, we identified a polymorphism A→G (rs3746444) in miR‐499 precursor (pre‐miR‐499) that affects the maturation of miR‐499‐5p and alters its antiapoptotic function by converting stable A‐U base pair to wobble G‐U base pair in pre‐miR‐499 secondary structure. Furthermore, our results showed that the concentrations of plasma miR‐499‐5p could be correlated with myocardial infarction (MI) and heart failure (HF) patients in comparison with control subjects and polymorphism rs3746444 in miR‐499 could influence its abundance in plasma. Finally, our results also showed that the variant of polymorphism in miR‐499 influenced the severity of the myocardial infarction significantly. This is the first report to highlight the biological significance of this polymorphism on the maturation of miR‐499‐5p and its antiapoptotic role during MI. These findings may pave a way to better understand the individual variability based on miRNA SNPs in heart diseases and may contribute to better treatment for disease severity on a personalized level.

## 1 INTRODUCTION

microRNAs (miRNAs) are a class of small non‐coding RNAs with 21‐25 nucleotides long and negative regulators of target genes by altering mRNA translation or stability.^1^, ^2^ It is estimated that miRNAs influence the expression of approximately 60% of human genes, and thereby, these small RNAs functionally participate in a wide variety of physiological or pathological processes.^3^, ^4^ miRNAs can regulate cardiac function such as the conductance of electrical signals, muscle contraction, heart growth and morphogenesis.^5^, ^6^, ^7^ There is a mounting evidence that miRNAs have been involved in the pathogenesis of cardiac diseases such as coronary heart disease (CHD) and heart failure (HF), and it may be possible to manipulate miRNA for therapeutic purpose.^8^

Single nucleotide polymorphisms (SNPs) in protein‐coding genes have been linked to different human diseases. miRNAs are transcribed from endogenous DNA and form hairpin structures (called pre‐miRNAs) that are processed to form mature miRNA. SNPs located in pre‐miRNA genes may directly affect the miRNA maturation, its expression or its binding to target mRNAs and, subsequently, alter the expression levels of target genes.^9^ Therefore, a functional SNP in miRNA genes may affect different signalling pathways by altering the expression levels of downstream genes. It has been shown that SNPs in miRNA genes of the individuals are associated with the susceptibility to many diseases, including cancer and cardiovascular disease.^10^, ^11^, ^12^, ^13^, ^14^ Previous studies reported that miR‐146a rs2910164, miR‐149 rs71428439, miR‐196a2 rs11614913 and miR‐499 rs3746444 might be related to the risk of heart diseases.^13^, ^15^, ^16^, ^17^ Studies have shown that the pre‐mir‐146a and pre‐mir‐149 polymorphisms alter their miRNA expression levels.^15^, ^18^ Previous reports showed that naturally occurring miR‐499‐5p mutation outside the critical seed sequence (miR‐499‐5p c17) modifies mRNA targeting and end‐organ function of miR‐499‐5p.^19^ However, it is not known whether the mechanism of genetic variants located in pre‐miR‐499 associated with the susceptibility to heart disease and whether pre‐mir‐499 rs3746444 affects the maturation and expression of miR‐499‐5p.

miR‐499‐5p is a cardiac‐abundant miRNA under physiological conditions.^20^, ^21^ It is regulated and functioned in heart development.^21^ Several studies revealed that miR‐499‐5p levels were down‐regulated under pathological conditions.^22^ Furthermore, miR‐499‐5p plays a cardioprotective role by inhibiting the apoptosis in cardiomyocytes and regulates the mitochondrial fission machinery.^22^, ^23^ miR‐499‐5p targets several pro‐apoptotic regulators such as programmed cell death protein 4 (Pdcd4), SOX6,calcineurin catalytic subunit α‐form (CnAα) and β‐form (CnAβ).^22^, ^23^, ^24^ In vivo studies suggested that miR‐499‐5p can inhibit myocardial infarction and cardiac remodelling upon ischaemic‐reperfusion,^22^ and its modulation may provide a therapeutic approach for treating myocardial infarction. Whether genetic variant in pre‐miR‐499 affecting its antiapoptotic function remains to be revealed.

Dysregulated miRNA expression patterns have been associated with various cardiac pathologies.^25^ Therefore, it may be considered that altered miRNA expression can be an indicator of heart damage that happened in pathological conditions. In particular, since the discovery of circulating miRNAs in the bloodstream, many studies are being performed to explore the association of miRNAs, expression levels and development of any particular disease to use miRNAs as diagnostic markers.^26^, ^27^ The previous reports have associated the elevated levels of miR‐499‐5p in plasma of subjects with acute myocardial infarction (MI) and below the detection levels in congestive HF. Therefore, the plasma levels of miR‐499‐5p may be helpful for distinguishing acute MI from cardiopathy in the cardiac patients.^28^ However, the field is still in its infancy and the mechanistic details of the differential expressions of miRNAs and its consequences are to be elucidated. Furthermore, whether SNPs located in these miRNAs may affect the sensitivity of plasma miRNAs, as diagnostic markers, is still uncovered.

Taking all these aspects into consideration, this study was aimed at exploring the mechanism that associates SNP rs3746444 of pre‐miR‐499 with the susceptibility of heart disease. Here, we report that the G allele miR‐499 precursor displays the decreased production of mature miR‐499‐5p compared with the A allele, which further affect the antiapoptotic function of miR‐499. We also found that plasma miR‐499‐5p can be used as diagnostic markers for MI; however, its genetic variants influence its levels in the plasma of patients. Therefore, the identification of the functional genetic variants in the miRNAs, which are associated with heart diseases, may improve the precision diagnostics of cardiac patients and improve our knowledge of their pathophysiology.

## 2 MATERIALS AND METHODS

### 2.1 Study subjects

All study subjects were recruited from Affiliated Hospital of Qingdao University, Qingdao, China. Approval for this study was obtained from the medical ethics committee of the Affiliated Hospital of Qingdao University. This study included 29 patients with myocardial infarction (MI group), 30 patients with chronic heart failure (HF group) and 14 asymptomatic controls (control group). Informed consent was obtained from all study subjects. Inclusion criteria for the HF group were chronic stable HF diagnosed according to Framingham standards, New York Heart Association Stage III‐IV and plasma prohormone of brain natriuretic protein (pro‐BNP) content ≥1000 ng/L. Inclusion criteria for the MI were based on the following criteria: (a) typical chest pain lasting longer than 30 minutes; (b) characteristic electrocardiographic patterns of myocardial infarction; (c) elevation of cardiac enzymes (creatine kinase and lactate dehydrogenase) and troponin I or T in blood. The control individuals were randomly selected from individuals without history of cardiovascular events and signs of dysfunction of the cardiovascular system by physical examination. These control subjects were frequency‐matched to the cases on age and sex. All subjects were genetically unrelated ethnic Han Chinese from Qingdao city and surrounding regions.

### 2.2 Genotyping

Genomic DNA was extracted from peripheral blood samples. Genotypes were analysed by polymerase chain reaction (PCR)‐based restriction fragment length polymorphism (RPLP) described.^13^ Briefly, the primers were:5′‐CAAAGTCTT CACTTCCCTGCCA‐3′ (forward); 5′‐GATGTTTAACTCCTCTCCACGTGATC‐3′ (reverse) for hsa‐mir‐499 rs3746444 amplification. The PCR product was digested with *Bcl 1* (NEB, Beverly, MA). DNA sequencing was performed for the confirmation for heterozygous genotypes.

### 2.3 RNA extraction

Blood samples were obtained from antecubital vein of each subject. After rapid centrifugation, plasma samples were transferred to RNase/DNase‐free tubes and stored at −80°C. Total RNA was isolated from 500 μL of plasma with TRIzol reagent (Invitrogen, Grand Island, NY). The plasma levels of miR‐499 were measured using a qRT‐PCR assay.

### 2.4 Construction of pri‐miR‐499 expression vectors

To create the allelic A and G pre‐miR‐499 expression vectors separately, the 600‐bp DNA fragments encompassing the miR‐499 precursor sequence and its 5‐ and 3‐flanking regions (281 and 197 bp, respectively) were amplified from human genomic DNA from subjects included in the study (determined to have the AA or GG genotype) and cloned into the XhoI and XbaI sites of the vector pcDNA3.1 (Invitrogen). The sequences of both vectors were confirmed by direct sequencing, and the only difference was in the site of SNP. The yielded vector with the AA genotype was designated as pri‐miR‐499‐A, and the one with the GG genotype was named as pri‐miR‐499‐G.

### 2.5 Quantitative real‐time PCR (qRT‐PCR)

Stem‐loop qRT‐PCR was carried out on a Roche Light Cycler® 480II. Total RNA was extracted using TRIzol reagent. RNA was reverse‐transcribed with reverse transcriptase kit (Takara, Otsu, Japan). Mature miR‐499‐5p level was measured using SYBR Green Real‐time PCR Master Mix (Takara) according to the manufacturer's instructions. The sequences of miR‐499‐5p primers were: forward, 5′‐ TTAAGACTTGCAGTGATGTTT‐3′; reverse, 5′‐GTGCAGGGTCCGAGGT‐3′. The sequences of miR‐499‐3p‐A primers were: forward, 5′‐ACACTCCAGCTGGGAACATCACAGCAAGTC‐3′; reverse, 5′‐ TGGTGTCGTGGAGTCG‐3′. The sequences of miR‐499‐3p‐G primers were: forward, 5′‐ACACTCCAGCTGGGAACGTCACAGCAAGTC‐3′; reverse, 5′‐ TGGTGTCGTGGAGTCG‐3′. The levels of miR‐499‐5p were normalized to that of miR‐16‐5p for plasma samples as described previous.^29^, ^30^ The sequences of miR‐16‐5p primers were: forward, 5′‐ TAGCAGCACGTAAATATTGGCG ‐3′; reverse, 5′‐ GTGCAGGGTCCGAGGT ‐3′. As for cell samples, miRNA expression was normalized to U6. The sequences of U6 primers were: forward, 5′‐GCTTCGGCAGCACATATACTAA‐3′; reverse, 5′‐AACGCTTCACGAATTTGCGT‐3′. For quantitative detection of pri‐miR‐499 and pre‐miR‐499, qRT‐PCR was performed as described.^22^

### 2.6 Cell culture, treatment and TUNEL analysis

H9c2 cardiomyocytes, a foetal rat cardiomyocyte‐derived cell line (American Type Culture Collection) were cultured in Dulbecco's modified Eagle's medium (Gibco, Grand Island, NY) supplemented with 10% foetal bovine serum, 100 U/mL penicillin, 100 g/mL streptomycin and 110 mg/mL sodium pyruvate in a humidified atmosphere containing 5% CO_2_ at 37°C. Cells were treated with 100 μmol/L H_2_O_2_ for indicated times. TUNEL assay was performed with a kit from Roche Applied Science (Hamburg, Germany). The procedures were following the kit instructions. The samples were imaged using a laser scanning confocal microscope (Zeiss LSM 510 META).

### 2.7 Cell transfection with miRNA duplexes or miRNA inhibitors

The miR‐499‐5p duplexes were synthesized by GenePharma Co. Ltd (Shanghai, China). miR‐499‐5p mimic sense sequence was, 5′‐UUAAGACUUGCAGUGAUGUUU‐3′. Mimic control sequence was, 5′‐UUCUCCGAACGUGUCACGUTT‐3′. Chemically modified antisense oligonucleotides (antagomirs) were used to inhibit endogenous miR‐499‐5p expression (GenePharma Co. Ltd). The antagomir sequence was, 5′‐AAACAUCACUGCAAGUCUUAA‐3′. All the bases were 2′‐O‐methyl‐modified. The antagomir control sequence was, 5′‐CAGUACUUUUGUGUAGUACAA‐3′. Cells were transfected with miRNA duplexes (100 nmol/L) or antagomirs (100 nmol/L) using Lipofectamine 2000 (Invitrogen, Grand Island, NY) according to the manufacturer's instructions.

### 2.8 Immunoblotting

Immunoblotting was performed as described previously.^31^ Briefly, cells were lysed for 1 hour at 4°C in a lysis buffer containing a protease inhibitor cocktail. Protein samples were subjected to 12% SDS‐PAGE and transferred to nitrocellulose membranes. Blots were probed using corresponding primary antibodies. Then the horseradish peroxidase‐conjugated secondary antibodies were used. Antigen‐antibody complexes were tested by enhanced chemiluminescence. The antibodies to CnAα and CnAβ were purchased from Millipore.

### 2.9 Reporter constructions and luciferase assay

Reporter vectors bearing miR‐499 binding sites of 3′ UTRs of CnAα and CnAβ were cloned into the pGL3 vector (Promega) immediately downstream of the stop codon of the luciferase gene as described previously.^22^ For luciferase assay, HEK‐293 cells in 24 well plates were cotransfected with the plasmid constructs of 200 ng/well of pGL3‐CnAα‐3′ UTR or pGL3‐CnAβ‐3′ UTR, 400 ng/well of miR‐499‐A or miR‐499‐G plasmid. pRL‐TK vector containing *Renilla* luciferase cDNA (5 ng/well) served as the internal control. pcDNA3.1 plasmid served as a negative control. 48 hours after transfection, cells were lysed and luciferase activity was measured with the dual luciferase kit (Promega, Madison, WI).

### 2.10 Statistical analysis

The results are expressed as means ± SD of at least three independent experiments. The differences among experimental groups were evaluated by one‐way analysis of variance for comparison among three or more groups and the two‐tailed *t* test applied for the comparison between two groups, respectively. *P* < 0.05 was considered statistically significant. For miRNAs with significant differences and associations, the ability to discriminate between the MI and control groups was characterized by the receiver operating characteristic (ROC) curve, and the area under the ROC curve (AUC) was estimated to assess the diagnostic accuracy of miRNAs. Statistical analyses were performed with SPSS 13.0. ROC curve was evaluated with GraphPad Prism 5.

## 3 RESULTS

### 3.1 The polymorphism rs3746444 changes A‐U pair to G‐U pair in miR‐499

**Figure 1:**
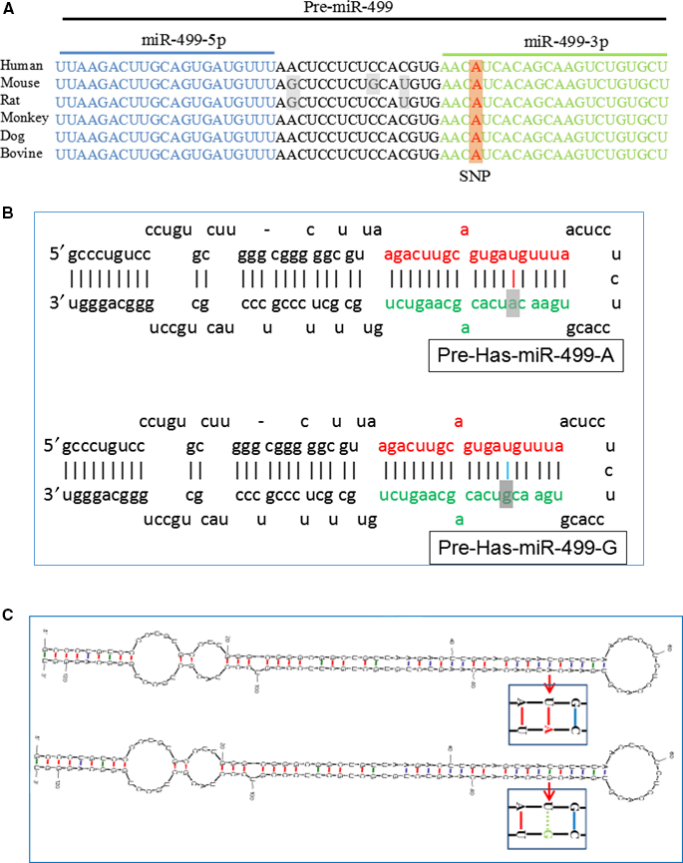
The rs3746444 polymorphism changes the conserved U‐A Watson‐Click base pair to U‐G wobble pair in pre‐miR‐499. A, Multiple sequence alignment of pre‐miR‐499 from mammals. B, Hairpin loop structure of the A‐ and G‐allelic miR‐499 precursor. The A→G polymorphism located in the stem region within the mature miR‐499‐3p sequence which is indicated by the dark colour. C, Secondary structure of pre‐miR‐499‐A and pre‐miR‐499‐G predicted by mfold web server. The polymorphism site changes the U‐A Watson‐Click base pair to U‐G wobble pair in pre‐miR‐499

The miR‐499 gene is located within intron 20 of human cardiac β‐myosin heavy chain 7B gene (MHC7b) in 7q11.^21^ miR‐499‐5p and miR‐499‐3p are derived from the 5′ and 3′ arms of pre‐miR‐499 hairpin precursor, respectively. miR‐499‐5p is the guide strand of miR‐499 gene and highly expressed in cardiac tissue under physiological conditions.^21^ The polymorphism, rs3746444, is located at the 73rd position of pre‐miR‐499 (the 6th position of miR‐499‐3p). Therefore, it is referred as miR‐499 A73G SNP. Alignment of available pre‐miR‐499 sequences revealed that miR‐499 SNP, rs3746444, is highly conserved in mammals (Figure 1A). The nucleotide C73 is located in the stem of pre‐miR‐499 and forms an A‐U base pair with nucleotide U49 (Figure 1B). The A73G polymorphism changes the conserved A‐U base pairs to G‐U wobble pairs (Figure 1B). We used mfold web server 32 to predict the minimum free energy for the secondary structure of pre‐miR‐499‐A and pre‐miR‐499‐G to detect the effect of this variant on the secondary structure of pre‐miR‐499. We did not observe a significant change in the minimum free energy by altering this nucleotide A to G (Figure 1C). Although this polymorphism did not affect secondary structure of pre‐miR‐499, the alteration of stable A‐U base pair to wobble G‐U base pair could potentially affect miR‐499 maturation, including primary miR‐499 (pri‐miR‐499) processing by Drosha, pre‐miR‐499 processing by Dicer and/or miR‐499 duplex stability.

### 3.2 The polymorphism rs3746444 affects the maturation of miR‐499‐5p

**Figure 2:**
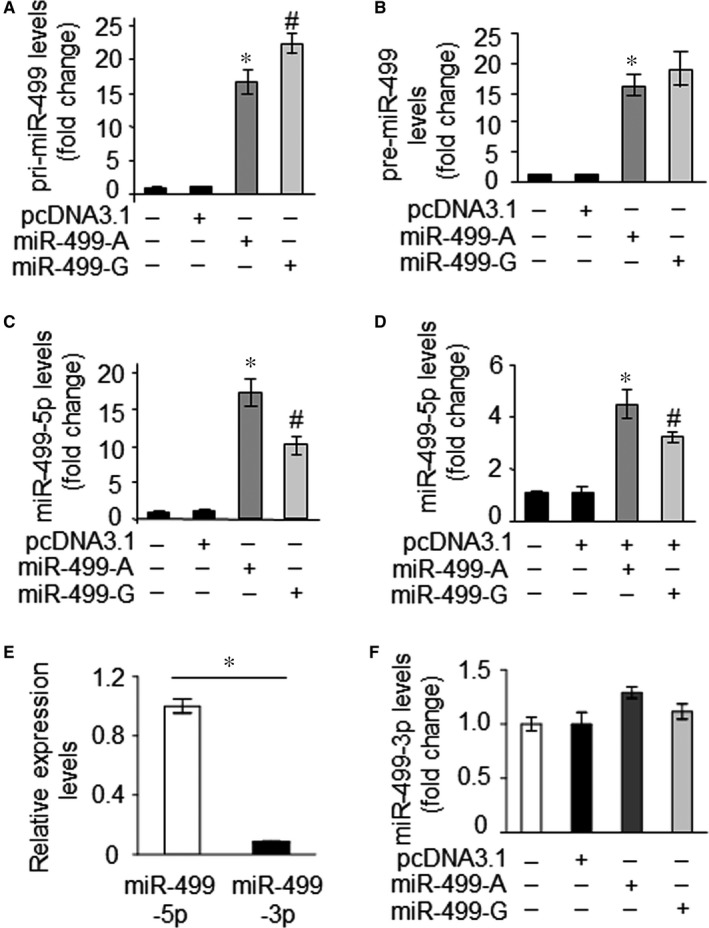
The rs3746444 polymorphism affects the maturation of miR‐499‐5p. A, pri‐miR‐499 and pre‐miR‐499 B, are not affected by this SNP. HEK‐293 cells were transfected with the plasmid of empty pcDNA3.1, pri‐miR‐499‐A or pir‐miR‐499‐G, respectively. Twenty‐four hours after transfection, pri‐miR‐499 and pre‐miR‐499 were analysed by qRT‐PCR as described in Materials and Methods. *P <* *0.05 vs empty plasmid. C, G allele shows a low level of mature miR‐499‐5p compared with the A allele in HEK‐293 cells. HEK‐293 cells were transfected with the plasmid as above, and miR‐499‐5p was analysed by qRT‐PCR. *P <* *0.05 vs empty plasmid, #*P *<* *0.05 vs miR‐499‐A. D, G allele impairs miR‐499 maturation in H9c2 cardiomyocytes. H9c2 cells were transfected with the plasmid of empty pcDNA3.1, pri‐miR‐499‐A or pri‐miR‐499‐G, respectively. Twenty‐four hours after transfection, miR‐499‐5p was analysed by qRT‐PCR. *P <* *0.05 vs empty plasmid, #*P *<* *0.05 vs miR‐499‐A. Data are expressed as the mean ± SD of 3 independent experiments. E, miR‐499‐5p and miR‐499‐3p levels were detected in H9c2 cells. *P <* *0.05. F, miR‐499‐3p level is not affected by this SNP. HEK‐293 cells were transfected with the plasmid of empty pcDNA3.1, pri‐miR‐499‐A or pir‐miR‐499‐G, respectively. Twenty‐four hours after transfection, miR‐499‐3p was analysed by qRT‐PCR as described in Materials and Methods. Data are expressed as the mean ± SD of 3 independent experiments

To explore whether the presence of the polymorphism rs3746444 in pre‐miR‐499 affects production of miR‐499‐5p, miR‐499‐5p abundance was quantified. The expression level of endogenous miR‐499‐5p was low in HEK‐293 cells. HEK‐293 cells were transfected with pcDNA3.1‐pri‐miR‐499‐A (pri‐miR‐499‐A) or pcDNA3.1‐pri‐miR‐499‐G (pri‐miR‐499‐G) or pcDNA3.1 empty vector. To examine the transcription of miR‐499 in HEK‐293T cells, we quantified pri‐miR‐499 transcripts in total RNA obtained from HEK‐293 cells by quantitative real‐time PCR (qRT‐PCR). We observed similar levels of pri‐miR‐499 in both the miR‐499‐A and the miR‐499‐G plasmids transfected cells, indicating that the polymorphism (A73G) did not affect the transcription of miR‐499 (Figure 2A). We also found the level of pre‐miR‐499 did not affect by this polymorphism (Figure 2A,B). Next, we quantified the abundance of mature miR‐499‐5p by qRT‐PCR. In contrast, the A73G SNP markedly reduced the levels of mature miR‐499‐5p in HEK‐293 cells (Figure 2C). Furthermore, the expression levels of miR‐499‐5p were also altered between different alleles in H9c2 cardiomyocytes transfected with above plasmid. The abundance of mature miR‐499‐5p was reduced in the G allele plasmids transfected cells in comparison with A allele (Figure 2D). miR‐499‐5p is the dominant mature miRNA product of miR‐499 gene as shown in the microRNA database miRBase. Whether the polymorphism rs3746444 affects miR‐499‐3p level is unknown. We detected miR‐499‐3p level in cardiomyocytes. Comparing to miR‐499‐5p,the level of miR‐499‐3p in cardiomyocytes is rather low (Figure 2E). Next, HEK‐293 cells were transfected with pri‐miR‐499‐A or pri‐miR‐499‐G and miR‐499‐3p level was detected. However, miR‐499‐3p level was not significantly affected (Figure 2F). It indicated that miR‐499‐3p is rarely expressed and not affected by this SNP. These data suggested that the A to G substitution in pre‐miR‐499 affects the maturation of miR‐499‐5p. We concluded that the miR‐499 SNP reduces levels of miR‐499‐5p, without affecting the expression levels of pri‐miR‐499.

### 3.3 The polymorphism rs 3746444 affects antiapoptotic function of miR‐499‐5p

**Figure 3:**
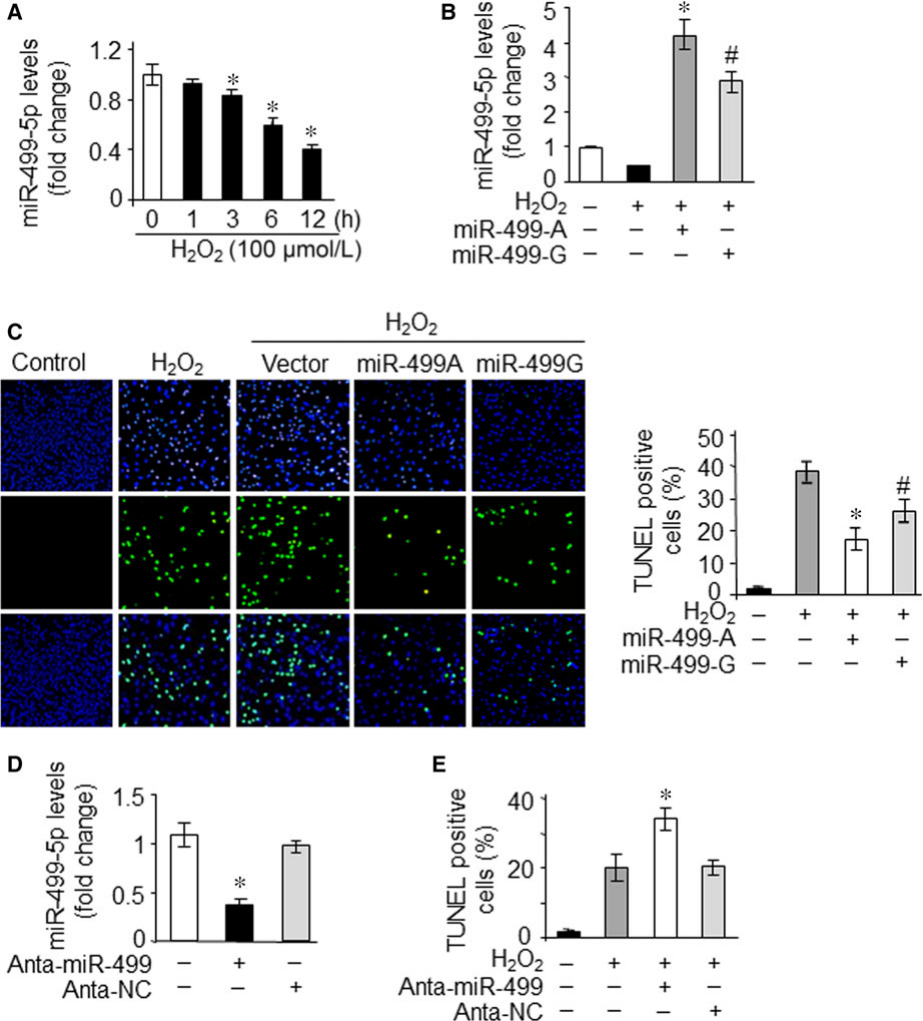
The rs3746444 polymorphism affects antiapoptosis function of miR‐499‐5p. A, miR‐499‐5p levels were detected in H9c2 cells exposed to 100 μmol/L H_2_O_2_ at the indicated time. *P <* *0.05 vs control. B and C, G‐allelic miR‐499 precursor inhibited apoptosis at a lesser degree compared with the A‐allelic one. H9c2 cells were transfected with the plasmid of pri‐miR‐499‐A or pri‐miR‐499‐G, respectively. After 24 hours, cells were exposed to 100 μmol/L H_2_O_2_ for 24 hours. miR‐499‐5p levels (B) and apoptosis (C) were analysed. Representative photographs showed TUNEL‐positive cell (C, left). TUNEL‐positive myocyte nuclei are green. Nuclei stained by DAPI show blue. The percentage of cells undergoing apoptosis were counted (C, right). *P <* *0.05 vs H_2_O_2_ alone, #*P *<* *0.05 vs miR‐499‐A. D and E, Knockdown of miR‐499‐sensitized H9c2 cells to apoptosis induced by H_2_O_2_. H9c2 cells were transfected with miR‐499‐5p antagomir (Anta‐miR‐499) or antagomir control (Anta‐NC). miR‐499‐5p levels were analysed 24 hours after transfection. *P <* *0.05 vs control. Cells were exposed to 50 μmol/L H_2_O_2_ for 24 hours, and apoptosis was analysed (E). *P <* *0.05 vs H_2_O_2_ alone. Data are expressed as the mean ± SD of 3 independent experiments

In our previous study, we have reported that miR‐499‐5p could inhibit apoptosis and myocardial infarction.^22^ H_2_O_2_ is well known ROS generating compound, which can induce apoptosis. Therefore, the expression levels of miR‐499‐5p in response to H_2_O_2_ treatment of H9c2 cardiomyocytes were explored. The expression levels of miR‐499‐5p were significantly down‐regulated in H9c2 cells exposed to 100 μmol/L H_2_O_2_ in a time‐dependent manner (Figure 3A). To test whether the variants of polymorphism (rs3746444) in miR‐499 may have any effect on its antiapoptotic ability, H9c2 cells were transfected with an equal amount of pri‐miR‐499‐A or pri‐miR‐499‐G plasmids, respectively, and treated with H_2_O_2_ to induce apoptosis. The significant up‐regulation of mature miR‐499‐5p was observed in response to the treatment (Figure 3B). Although both pri‐miR‐499‐A and pri‐miR‐499‐G markedly decreased the apoptosis, induced by H_2_O_2_, however, miR‐499‐G‐allelic variant showed a weaker apoptotic effect in comparison with miR‐499‐A allele (Figure 3C). Then, an antagomir targeting miR‐499‐5p was used to knock down endogenous miR‐499‐5p in H9c2 cells (Figure 3D). The knockdown of miR‐499‐5p sensitized H9c2 cells to apoptosis induced by 50 μmol/L H_2_O_2_ (Figure 3E). Therefore, these result suggested that the polymorphism rs 3746444 affects antiapoptotic function of miR‐499‐5p.

### 3.4 The polymorphism rs3746444 reduces the suppression of miR‐499‐5p target

**Figure 4:**
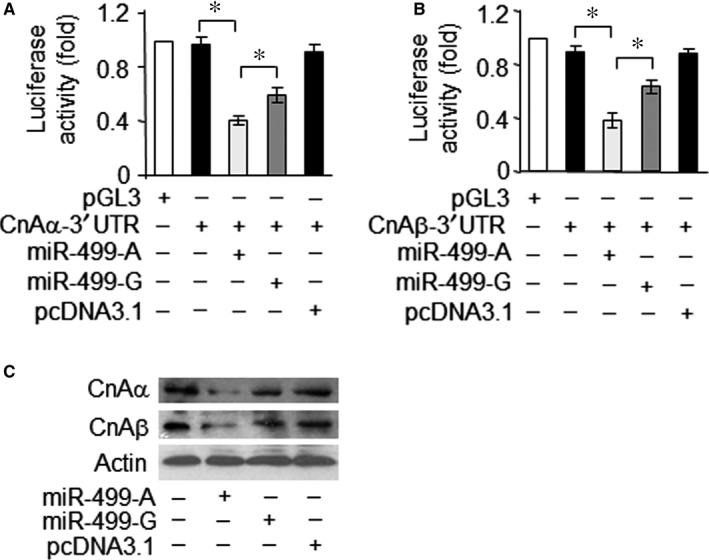
The rs3746444 polymorphism reduces target suppression by miR‐499‐5p. A and B, G‐allelic pre‐miR‐499 causes a lesser inhibition of *CnA*α‐3′UTR and *CnA*β‐3′UTR translation activity than the A‐allelic one. HEK293 cells were transfected with the luciferase constructs of *CnA*α‐3′UTR (A) or *CnA*β‐3′UTR (B), along with the plasmids for empty pcDNA3.1, pri‐miR‐499‐A or pri‐miR‐499‐G, respectively. Luciferase activity was measured after 36 hours. pGL3 served as a control. Data are expressed as the mean ± SD of 3 independent experiments. *P <* *0.05. C, G‐allelic pre‐miR‐499 suppresses the expression of *CnA*α and *CnA*β at a lesser degree than the A‐allelic one. H9c2 cells were transfected with the plasmids for empty pcDNA3.1, pri‐miR‐499‐A or pir‐miR‐499‐G, respectively. Twenty‐four hours after infection, *CnA*α and *CnA*β protein levels were detected by immunoblot. A representative blot of three independent experiments is shown

As the A73G SNP reduced the levels of miR‐499‐5p, we expected that the SNP might affect the target suppression of miR‐499‐5p. We reported previously that both isoforms of calcineurin catalytic subunit, the α‐form (CnAα) and β‐form (CnAβ) are the targets of miR‐499‐5p.^22^ Therefore the effect of pri‐miR‐499‐A and pri‐miR‐499‐G‐allelic variants on the translation of CnAα and CnAβ was tested by luciferase reporter assay. Cotransfection of CnAα 3′‐UTR reporter plasmid with pri‐miR‐499‐A plasmid resulted in significant decrease in the luciferase activity. However, the target suppression ability of the pri‐miR‐499‐G allele was significantly weaker than the pri‐miR‐499‐A allele by comparing their luciferase activities (Figure 4A). Similarly, cotransfection of CnAβ 3′‐UTR reporter plasmid with pri‐miR‐499‐A plasmid resulted in a marked decrease in luciferase activity, but suppression of the pri‐miR‐499‐G allele plasmid was again weaker for this target than miR‐499‐A‐allelic plasmid (Figure 4B). The overexpression of miR‐499‐5p led to an obvious reduction in both of its targets, CnAα and CnAβ. However, the reduction in the expression levels of both targets was significantly stronger in the presence of miR‐499‐A allele than miR‐499‐G allele (Figure 4C). This evidence suggested that the miR‐499 SNP reduces the suppression of miR‐499‐5p target, which is consistent with decreased expression levels of miR‐499‐5p due to the presence of this polymorphism.

### 3.5 Plasma miR‐499‐5p serves as a diagnostic marker for heart disease

**Figure 5:**
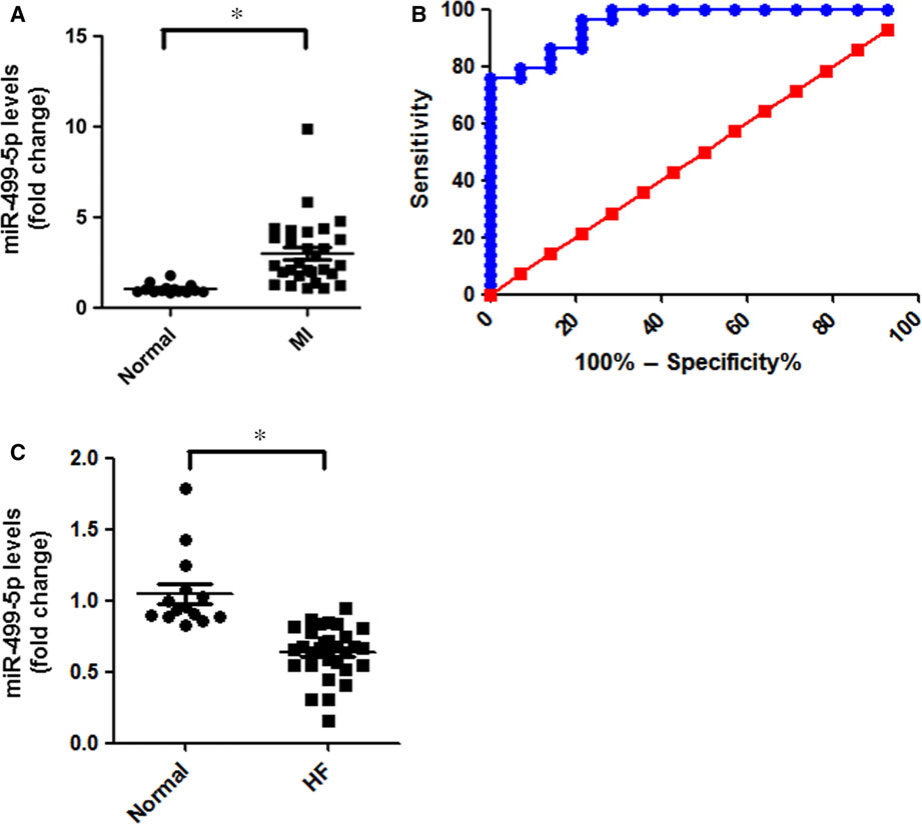
Plasma concentrations of miR‐499‐5p in the study population. A, miR‐499‐5p levels in plasma of patients with MI and controls were detected by qRT‐PCR. *p <* *0.05. B, The ability of miR‐499‐5p to discriminate the MI group from the control group. ROC curve analysis of miR‐499‐5p to distinguish between MI and control groups. C, miR‐499‐5p levels in plasma of patients with HF and controls were detected by qRT‐PCR. *P <* *0.05

The circulating miRNAs are being assessed as diagnostic and prognostic biomarkers because the altered levels of some miRNAs have been reported in human body fluids for different cardiac diseases.^33^ Plasma miR‐499‐5p has also been suggested as a biomarker of acute MI and CHD previously.^28^, ^34^, ^35^ We assessed the plasma concentrations of miR‐499‐5p in 29 individuals with acute MI, 30 individuals with congestive HF and 14 normal individuals without any cardiovascular diseases. The levels of plasma miR‐499‐5p concentrations between patients with MI or HF and control subjects were compared, respectively, using qRT‐PCR. The plasma miR‐499‐5p concentrations were higher in patients with MI in the acute phase (within 24 hours of the last onset of chest pain), with a median score of 2.364 than control subjects, with a median score of 0.941 (Figure 5A). The diagnostic accuracy of miR‐499‐5p was evaluated using receiver operating characteristic (ROC) curve analyses, miR‐499‐5p distinguished MI cases from healthy controls with an area under the curve (AUC) of 0.9557 (95% confidence interval, 0.90‐1.01; Figure 5B). Using a threshold score of 1.59 above which patients are predicted to belong to MI group, we achieve a sensitivity of 79.31% and a specificity of 92.86% for identification of MI patients. In contrast, plasma miR‐499‐5p concentrations were decreased in patients with HF, with a median score of 0.664 in the HF group, than the control subjects, with a median score of 0.941 (Figure 5C).

### 3.6 The allelic variants of polymorphism, rs3746444, affect the plasma levels of miR‐499‐5p and its clinical features in MI patients

**Figure 6:**
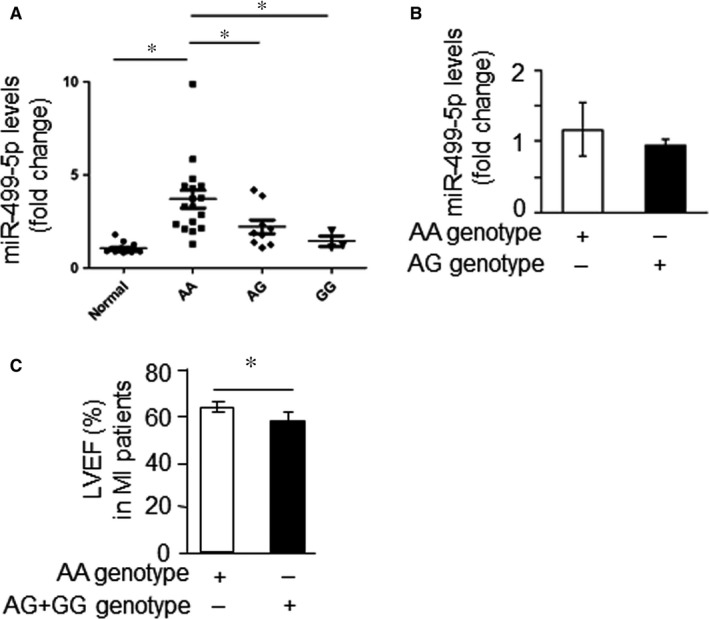
The rs3746444 polymorphism affects the level of plasma miR‐499‐5p and clinical features of MI patients. A, Analysis of plasma miR‐499‐5p levels in MI patients based on miR‐499 rs3746444 genotype. *P <* *0.05. B, Analysis of plasma miR‐499‐5p levels of normal people based on miR‐499 rs3746444 genotype. In this sample, 6 people carry AA genotype, and 7 people carry AG genotype. C, Analysis of cardiac function of MI patients based on miR‐499 rs3746444 genotype. *LVEF*, left ventricular ejection fraction. *P <* *0.05

The polymorphism rs3746444 significantly influencing the maturation of miR‐499‐5p motivated us to further study whether this SNP genotype affects the level of plasma miR‐499‐5p and clinical features for MI patients. The levels of miR‐499‐5p were statistically different between MI patients with AA genotype compared to those with AG or GG genotypes. As shown in Figure 6A, the plasma miR‐499‐5p levels of MI patients with AA alleles increased about 3.69‐fold compared with normal population, which was significantly higher than the other two alleles AG (2.2 folds) and GG (1.43 folds). While in normal population, the level of plasma miR‐499‐5p was not affected by this SNP genotype. (Figure 6B). However, the sample size was small, it need to be further verified. Therefore, it can be inferred that patients with AA genotype of miR‐499 are most sensitive to MI diagnosis in comparison with AG and GG genotype.

We further compared the clinical feature of MI patients based on the miR‐499 SNP genotype. It was observed that left ventricular ejection fraction (LVEF) in patients carrying the G allele in the genome was significantly lower than that of patients carrying the A allele (Figure 6C). Taken together, these results suggested that the polymorphism rs3746444 could significantly influence the severity of heart disease and diagnostic sensitivity of MI when considering miR‐499‐5p as a biomarker for disease detection.

## 4 DISCUSSION

The human genome is enriched with polymorphisms that result in different phenotypes. Accumulating evidence suggests that miRNAs play important regulatory roles in different diseases including cardiovascular disease.^25^ The polymorphisms in miRNAs have been linked to susceptibility to cardiovascular diseases such as myocardial infarction and coronary heart disease.^13^, ^16^, ^36^ The SNPs in miRNA genes may influence the processing and/or target selection of miRNAs.^15^ In this study, we first identified that SNP rs3746444 located in miR‐499 precursor can affect the expression levels and antiapoptotic function of miR‐499‐5p, which have been associated with MI and HF. We also observed that the individual's genotype of the SNP rs3746444 alters the diagnostic sensitivity of circulating miR‐499‐5p marker for the cardiovascular diseases.

Although the association between the SNPs in protein‐coding genes and the risk of many kinds of diseases has been extensively investigated, there are only a few reports about the SNPs in miRNAs which are associated with risk of cardiovascular diseases. Previous studies have shown that several miRNA polymorphisms (miR‐146a rs2910146, miR‐149 rs71428439, miR‐499 rs37464444, miR‐208 rs11134527 and miR‐149 rs71428439) contribute to the risk for the development of cardiovascular diseases including CHD, HF and MI.^13^, ^15^, ^18^, ^36^ The future studies are required to further explore the roles of SNPs in miRNA related to the risk of other cardiovascular diseases including cardiomyopathy.

As miR‐499‐5p is highly expressed in cardiac tissue and participates in the maintenance of cardiac survival and function,^21^, ^25^ it is reasonable to propose that polymorphism rs3746444 may contribute to the susceptibility of heart diseases. For the polymorphism rs3746444 (A→G) of hsa‐mir‐499, the few previous studies have shown that the variant genotype GG of this SNP was significantly associated with the increased risk of cancer^37^ and dilated cardiomyopathy,^38^ but most of the studies found no significant association with the increased risk of cancer.^39^, ^40^, ^41^ It has been revealed that the GG and AG genotypes of SNP rs3746444 conferred increased risk for CHD 13 and ischaemic stroke,^42^ respectively. Furthermore, the GG genotype increased the risk of MI in a Chinese population,^16^ indicating that MI may share genetic risk factor with both CHD and ischaemic stroke. However, Xiong et al^18^ found no association between SNP rs3746444 and CHD. One potential explanation for this phenomenon may be different genetic backgrounds among races.

The possible mechanism for such associations might be due to impaired maturation of miR‐499‐5p in GG homozygotes in comparison with AA homozygotes. The variation of a single base in the genes of miRNAs is able to influence maturation of miRNAs or alters their target sites, ultimately results in the altered activities of miRNAs.^5^, ^9^, ^15^, ^43^ The A→G polymorphism (rs3746444) is located in the stem region outside the mature miR‐499‐5p sequence. We observed that G‐allelic miR‐499 precursor displayed a low efficiency to produce mature miR‐499‐5p compared with the A‐allelic one. miR‐499 has been shown to suppress the apoptosis through targeting the pro‐apoptotic protein CnAα and CnAβ. The A to G variation due to the polymorphism in the miR‐499 precursor led to a reduced inhibiting effect of pri‐miR‐499 on the expression of CnAα/CnAβ and consequently exhibited a less repressive effect on the apoptosis. The “loss of function effect” of miR‐499 A→G SNP on the maturation of miR‐499‐5p and its abundance is most likely due to the replacement of A‐U Watson‐Click base pair with the pre‐miR‐499 stem and miR‐499 duplex with a G‐U wobble base pair. There is a possibility that this change in the base may disrupt the geometry of the RNA helix and thus may affect the recognition of pri‐ and/or pre‐miR‐499 stem and miR‐499 duplex by enzymes or RNA‐binding proteins. Another study has shown that miR‐590 C→T SNP (rs6971711) also replaces the stable C‐G base pair with wobble U‐G base pair and affected the maturation of miR‐590 from pre‐miR‐590.^44^ Furthermore, SNPs in the seed region of miRNAs might reduce or even lose their interacting ability with the 3′ UTR of target mRNAs and hence reduce their role of target suppression.^43^ Oppositely, it might also change the targets of miRNAs due to increased binding affinity for new target sites.^45^, ^46^ Our findings suggested that SNPs could influence the level of miRNA, resulting in the change of miRNA targets' expression levels. Other studies have also suggested that the sequence variations caused by SNPs or mutations may greatly contribute to the heterogeneity of complex diseases. However, the exact mechanism is still not clear. Therefore, further studies are required to identify the exact mechanism by which SNP can influence the miRNA expression, its processing, maturation and/or its downstream effects on target genes.

Accumulating evidence suggests that the circulating miRNAs can be used as stable blood‐based biomarkers for various diseases.^26^, ^27^ The cardiac‐specific or enriched miRNAs such as miR‐1, miR‐133, miR‐499‐5p and miR‐208a have been found to be elevated in the bloodstream of acute MI patients.^34^, ^47^ These findings highlight the potential value of miRNAs as novel biomarkers for the diagnosis and prognosis of MI. Here, we found that plasma miR‐499‐5p concentrations were increased in patients with MI in the acute phase compared with age‐matched normal control subjects. However, the circulating miR‐499‐5p concentrations were lower in heart failure patients in comparison with normal healthy individuals. Our results indicated that plasma miR‐499‐5p concentration could be a biomarker of MI in humans, which is consistent with previous findings. We also found the SNP rs3746444 affects the maturation of miR‐499‐5p and the levels of plasma miR‐499 were significantly higher in patients carrying A‐allelic variant than G allele. As patients carrying A‐allelic variant have higher cardiac miR‐499‐5p level than G allele, we can infer that when they suffered from MI, more miR‐499‐5p would be released to plasma from the damaged cardiomyocytes. Therefore, it is suggested that care must be taken for the presence of any SNPs in miRNAs before considering miRNAs for diagnostic markers as SNP may influence the sensitivity of miRNAs for diagnostic purpose. Hence, multicenter and large cohort studies are required to confirm their suitability as a biomarker of MI or any other human diseases. Moreover, serum miRNA has not been widely practiced in clinical application, the diagnosis techniques for quick and accurate circRNA detection need to be validated.

However, it is debatable where the circulating miRNA of bloodstream might originate from in cardiac patients. MI is characterized by cardiac injury under ischaemic and hypoxic stress that results in the release of various proteins from damaged cardiac cells into the circulation, including cardiac troponin T and creatine kinase (CK). It is highly likely that the heart tissue damage may cause an additional release of miRNA similar to the release of proteins. The secretion of miRNAs can also occur via small vesicles arising from the plasma membrane or endosomes and are constantly released by almost all types of cells.^48^ Another likely source might be platelets that release their miRNA content into the circulation upon activation.^49^, ^50^ miR‐499‐5p is a cardiac‐specific miRNA and serum miR‐499‐5p levels are increased at an early time of cardiac injury. miR‐499‐5p levels were decreased in the plasma of heart failure patients, which may be due to down‐regulation of miR‐499‐5p in the heart under pathological conditions.^22^ These results suggested that elevation of miR‐499‐5p in plasma is may be a result of cardiomyocyte necrosis and subsequent release of cellular constituents into the circulating system in patients with acute MI. It seems that compared with cardiac‐enriched miRNAs, non‐cardiac miRNAs have a comparable potential as MI biomarker.^51^, ^52^ However, the origin and release of these miRNAs in circulation is an interesting query which needs to be investigated in future studies.

It has been reported that the presence of miRNAs in the extracellular space and circulation indicates that these molecules may exhibit signalling functions by acting in an autocrine, paracrine and possibly endocrine manner.^53^, ^54^ Although the several studies have assessed the functions of miRNAs in cell‐to‐cell or cell‐to‐surrounding‐environment communications, however, the consequences and biological effects of systemically released miRNAs on distant sites remain challenging questions. Furthermore, whether the circulating miR‐499‐5p involved in biological effect at distant sites or in organs or tissues other than the heart need to be further explored.

In summary, this study revealed that miR‐499‐5p polymorphism may affect its function by regulating apoptosis. Identifying more miRNA polymorphisms and exploring their mechanisms will greatly improve our knowledge of the aetiology of diseases and provide a useful tool for creating an efficient individualized treatment for the complicated diseases.

## CONFLICT OF INTEREST

The authors declare no conflict of interests.
